# Dual-Located WHIRLY1 Interacting with LHCA1 Alters Photochemical Activities of Photosystem I and Is Involved in Light Adaptation in Arabidopsis

**DOI:** 10.3390/ijms18112352

**Published:** 2017-11-07

**Authors:** Dongmei Huang, Wenfang Lin, Ban Deng, Yujun Ren, Ying Miao

**Affiliations:** Center for Molecular Cell and Systems Biology, Fujian Provincial Key Laboratory of Haixia Applied Plant Systems Biology, College of Life Sciences, Fujian Agriculture and Forestry University, Fuzhou 350002, China; 2130516002@fafu.edu.cn (D.H.); linwf@fafu.edu.cn (W.L.); 1140539002@fafu.edu.cn (B.D.); ryj@fafu.edu.cn (Y.R.)

**Keywords:** electron transport rate (ETR), light, photochemical activities, photosystem I, plastid gene, WHIRLY1

## Abstract

Plastid-nucleus-located WHIRLY1 protein plays a role in regulating leaf senescence and is believed to associate with the increase of reactive oxygen species delivered from redox state of the photosynthetic electron transport chain. In order to make sure whether WHIRLY1 plays a role in photosynthesis, in this study, the performances of photosynthesis were detected in *Arabidopsis whirly1* knockout (*kowhy1*) and plastid localized WHIRLY1 overexpression (*oepWHY1*) plants. Loss of WHIRLY1 leads to a higher photochemical quantum yield of photosystem I Y(I) and electron transport rate (ETR) and a lower non-photochemical quenching (NPQ) involved in the thermal dissipation of excitation energy of chlorophyll fluorescence than the wild type. Further analyses showed that WHIRLY1 interacts with Light-harvesting protein complex I (LHCA1) and affects the expression of genes encoding photosystem I (PSI) and light harvest complexes (LHCI). Moreover, loss of WHIRLY1 decreases chloroplast NAD(P)H dehydrogenase-like complex (NDH) activity and the accumulation of NDH supercomplex. Several genes encoding the PSI-NDH complexes are also up-regulated in *kowhy1* and the *whirly1whirly3* double mutant (*ko1/3*) but steady in *oepWHY1* plants. However, under high light conditions (800 μmol m^−2^ s^−1^), both *kowhy1* and *ko1/3* plants show lower ETR than wild-type which are contrary to that under normal light condition. Moreover, the expression of several PSI-NDH encoding genes and *ERF109* which is related to jasmonate (JA) response varied in *kowhy1* under different light conditions. These results indicate that WHIRLY1 is involved in the alteration of ETR by affecting the activities of PSI and supercomplex formation of PSI with LHCI or NDH and may acting as a communicator between the plastids and the nucleus.

## 1. Introduction

Chloroplasts serve as sensors of environmental conditions and guide plants toward proper adaption [1]. They can remodel the photosynthetic apparatus and maximize photosynthetic efficiency under various environmental conditions [2,3]. Photosynthetic light reactions are essential to the retrograde plastid-to-nucleus signaling which contributes to the coordination of chloroplast function by regulating nuclear gene expression [4,5,6]. Although novel possible plastidial signals including the transcription factor WHIRLY1 (WHY1) were proposed [7,8,9], the association of WHY1 and retrograde plastid-to-nucleus signaling remains elusive.

WHY1 is a plastids-nucleus bi-located protein and belongs to a small family of single-stranded DNA (ssDNA) binding proteins [10,11]. In monocotyledonous plants, the WHIRLY family contains two members, whereas in dicotyledonous plants, they have three members [12]. In *Arabidopsis*, WHY1 and WHY3 were discovered in the proteome of transcriptionally active chromosome (TAC), which is the transcriptionally active fraction of nucleoids [13]. Most of the double knockout mutants lacking both WHY1 and WHY3 (*why1why3*, *ko1/3*) had no difference in phenotype with the wild-type [14,15]. In contrast, the *why1why3polIb-1* triple mutant that is defective in WHY1 and WHY3 as well as the chloroplast DNA polymerase 1B (Pol1B) exhibited a more extreme yellow-variegated phenotype [16]. The *why1why3polIb-1* mutants showed lower photosynthetic electron transport efficiencies and higher accumulation of reactive oxygen species compared to wild-type plants [16]. The higher level of oxidation observed in this mutant was linked to chloroplast to nucleus signaling and enhanced the adaptation to oxidative stress [16]. WHY3 protein was identified as a redox-affected protein in chloroplasts, while WHY1 protein was indicated as a thioredoxin target [8]. Moreover, WHY1 was proposed to link the operation of the photosynthetic electron transport chain to gene expression due to its location that is at the boundary between thylakoids and nucleoids [8]. Therefore, WHY1 is important in the perception of redox functions within the electron transport system which is closely linked to photosynthesis [8].

Recently, a new research showed that young leaves of *WHY1* RNAi knockdown mutants (W1-1, W1-7, W1-9) had lower CO_2_ assimilation rates and epoxidation state (EPS) than that in wild-type barley under high light condition [17]. However, CO_2_ assimilation rates in senescent leaves of *WHY1* RNAi mutants were higher than in wild-type while EPS was similar in both genotypes [17]. Furthermore, the VAZ (violaxanthin, antheraxanthin, zeaxanthin) pool was elevated in both young and senescent leaves of *WHY1* RNAi knockdown mutants [17]. Hence, WHY1 should be associated with photosynthesis and the metabolism during both chloroplast development and senescence progress. The slowing down of light-dependent senescence processes but not of dark-induced senescence in *WHY1*-defcient barley plants indicates that WHY1 is involved in sensing and signaling of light intensity [17]. Moreover, transcriptome analyses showed that *WHY1* was significantly enriched in light-regulated gene cluster and plastid-regulated gene cluster, implying that WHY1 has a role in integration of light and plastid signaling [18]. In addition, WHY1 in barely were assumed to connect the plastid and nuclear genes encoding photosynthetic proteins during abiotic stress [19]. However, the detail role of WHY1 in both pathways remains unknown.

Performances of photosynthesis such as optimization of electron transport, carbon assimilation, and assimilate production are important at all stages of leaf development, especially during senescence when chloroplasts are dismantled and photosynthetic proteins are recycled for vegetative or reproductive development and in particular for grain filling [20]. Therefore, photosynthesis and leaf senescence are determinants of plant productivity [20]. In this report, roles of WHY1 on photosynthesis have been investigated using *Arabidopsis WHY1* knock out and overexpression plants [21]. The effects of WHY1 on photosynthetic performances and photosystem I encoding genes were determined at early senescence stage. Then we further searched for the WHY1 interacting protein in chloroplast and analyzed the function and structure of the NDH complex which formed a supercomplex with PSI in *WHY1* mutants. Moreover, to determine whether WHY1 is involved in light adaption via regulating photosynthesis, we tested the photosynthesis parameters and PSI-LHCI, NDH gene expression in *kowhy1* and *ko1/3* mutants under high light conditions. Our results suggest that WHY1 is involved in regulating the photosynthesis process during senescence and stress.

## 2. Results

### 2.1. Changes of Photosynthetic Performance in WHY1 Mutants

**Figure 1:**
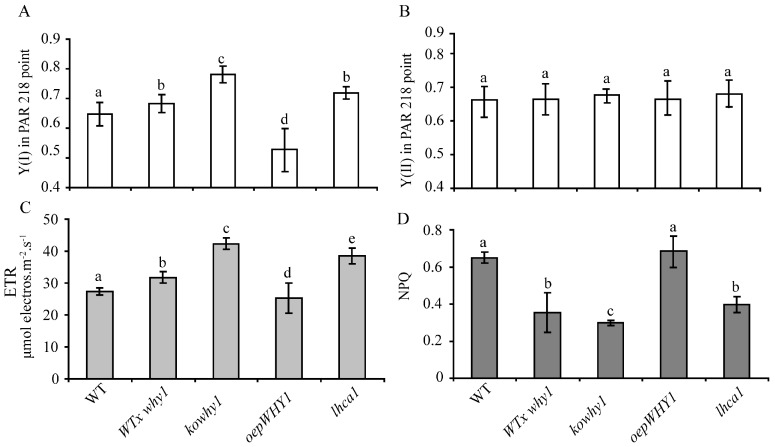
Analyses of the photosynthetic performance in leaves of the *WHY1*, *lhca1* mutants and the wild-type plants by a Dual PAM (Pulse-amplitude modulation) device. Dual PAM measurements of photochemical yield of photosystem I Y(I) (**A**), yield of photosystem II Y(II) (**B**), electron transport rate (ETR) (**C**), and photosystem II fluorescence (NPQ) (**D**), respectively. Data presented are mean ± SE (*n* = 6–8). Significance analysis was performed by Tukey–Kramer method (*p* < 0.05) and the data were dividing into different significance groups.

The function of the photosynthetic machinery can be probed by chlorophyll (chl) fluorescence emission by photosystem II (PSII) and light absorption by P700 reaction center chl a of photosystem I (PSI) [22]. To determine whether photosynthetic performance at leaf early senescence stage is affected by WHY1 in *Arabidopsis*, photosynthetic parameters in *WHY1* mutants were measured. The photochemical quantum yield of photosystem I Y(I) and photosystem II Y(II) was measured in fully expanded rosette leaves of different *WHY1* mutants at the seventh week by using a Dual PAM (Pulse-amplitude modulation) Measuring System (Walz, Effeltrich, Germany). Photosynthetic parameters in *Light-harvesting complex* *a* *1* knock out mutant (*lhca1*) that is defective in state transitions in photosynthesis were also measured [23]. The leaves were exposed to 125 μmol m^−2^ s^−1^ of actinic irradiance after dark adaptation for 15 min and transient 10,000 μmol m^−2^ s^−1^ of irradiance of the saturating pulses for 21 s. Y(I) is defined by the fraction of overall P700 that is reduced in a given state and is not limited by acceptor [24]. Y(II) is the efficiency of excitation capture by open PSII reaction centers. As showed in Figure 1A, Y(I) was higher in *kowhy1* plants than in wild-type, and in agreement, the Y(I) in the heterozygous line (WT*/why1*) created by back-crossing *kowhy1* with wild-type also slightly decreased but was still significantly higher than wild-type (Figure 1A), whereas Y(I) in *oepWHY1* plants that overexpressed *WHY1* in chloroplast were only about 60% of that in WT. Interestingly, the *lhca1* mutant line showed the same yield of Y(I) as the WT/*why1* heterozygous line (Figure 1). In addition, photosystem II yield was similar in wild-type and those *WHY1* mutants (Figure 1B). Therefore, loss of WHY1 seems to increase photosystem I quantum yield but not photosystem II quantum yield.

We also determined the nonphotochemical quenching of photosystem II fluorescence (NPQ) and the chloroplast electron transport rate (ETR) in *WHY1* and *lhca1* mutants. As showed in Figure 1D, though NPQ displayed a slight increase in the *overexpression plastid-localized WHY1* (*oepWHY1*) plants, it was clearly reduced in *WHY1* heterozygous knockout line (WT*/why1*) and homozygous line (*kowhy1*), and the latter showed a lower level (Figure 1D). In addition, NPQ was also lower in *lhca1* mutant compared to wild-type plants. Therefore, loss of WHY1 led to less excessive excitation energy production. The ETR in *kowhy1* mutants showed an increase of 35% than in wild-type, whereas the ETR was slightly decreased in *oepWHY1* plants (Figure 1C). Moreover, the heterozygous line (WT*/why1*) and *lhca1* mutant exhibited an intermediate ETR value between wild-type and the *kowhy1* mutant (Figure 1C). These results suggest that WHY1 acts as a repressor in electron transport progress.

### 2.2. WHY1 Affects the Expression of PSI-LHCI Encoding Genes

**Figure 2:**
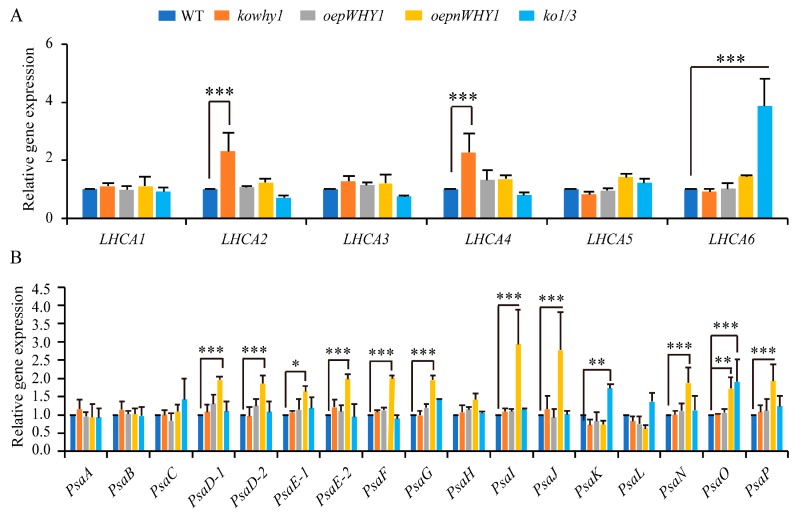
Expression of photosystem I (PSI)-light harvest complexes (LHCI) encoded genes in *WHY1* mutants and wild-type plants (**A**) Expression analysis of LHCI complex encoded genes in *WHY1* mutants and wild-type plants by qPCR; (**B**) Expression analysis of PSI core complex encoded genes in *WHY1* mutants and wild-type plants. The transcript level in each case was normalized to that of *GAPC2* as a reference gene, and the expression level of WT was set as 1. Three biological replications were used to analyze. Asterisk indicate significant differences in gene expression compared to WT (Tukey–Kramer method, * *p* < 0.05, ** *p* < 0.01, *** *p* < 0.001).

Previous transcriptome analyses showed that *WHY1* was significantly enriched in light-regulated gene cluster and plastid-regulated gene cluster [18]. Our previous microarray data analyses also showed that photosystem I related gene expression was significally up- or down-regulated in the inducible expression of *WHY1* or knockout of *WHY1* plants (Lin et al., 2017 manuscript). We wondered if WHY1 regulates the function and activity of photosystem I at transcriptional level. Therefore, we examined the expression of genes encoding reaction center and LHCI complex [25] under different *WHY1* mutant plants at 6th week by qRT-PCR when leaf senescence is initiated (Figure 2). *LHCA2* and *LHCA4*, both of which have been reported to be associated with the core in the absence of their “dimeric partners” [26], were up-regulated 2–4-fold in the absence of *WHY1* mutant (*kowhy1*), while genes encoding PSI core complex were not affected. Moreover, expression of *LHCA6*, *PsaK* and *PsaO* were significantly increased in *WHY1* and *WHY3* double mutant (*ko1/3*). In contrast, in the *overexpression plastid-localized WHY1* (*oepWHY1*) plants, which accumulated WHY1 only in plastids, did not alter the transcription levels of PSI or LHCI encoding genes. Interestingly, a large number of genes encoding the PSI core complex were up-regulated in both the nucleus and plastids overexpressed *WHY1* plants (*oepnWHY1*) (Figure 2B), whereas the transcription level of *LHCA1*-*6* was steady (Figure 2A). Since most of the PSI core subunit are critical to maintain the formation and functions of PSI [27], these results indicate that WHY1 is involved in regulating the expression of PSI encoding genes, but may not be a direct upstream regulator on plastid transcriptional level.

### 2.3. WHY1 Interacts with LHCA1 In Vitro and In Vivo

**Figure 3:**
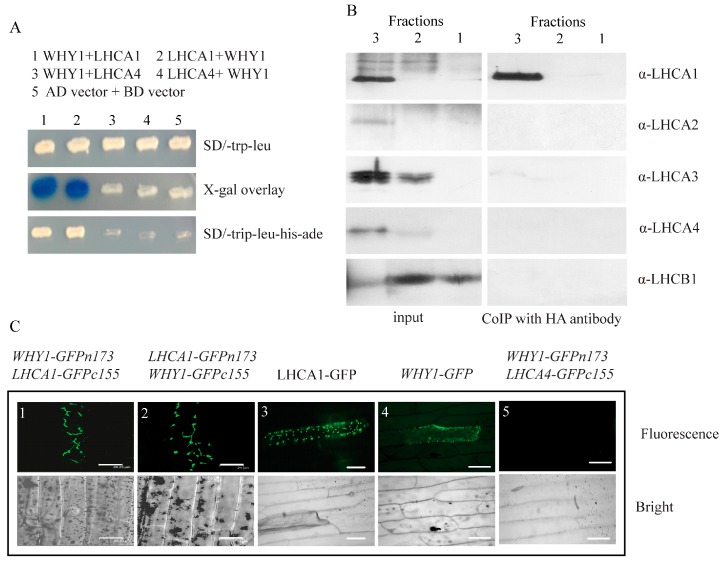
The interaction confirmation of WHY1 and LHCA1. (**A**) The interaction confirmation of WHY1 and LHCA1 by yeast two hybrid. Yeast two hybrid interaction detection growth selections and X-Gal agarose overlay assay indicate an interaction between WHY1 and LHCA1; (**B**) CoIP of WHY1 and LHCA1 from photosystem I containing thylakoid membrane complexes. As input to the thylakoid membrane fractions 1, 2, and 3 (see Supplementary Figure S1, 1, free pigments; 2, LHCII and photosystem II complexes; 3, photosystem I complexes) were used. Coimmunoprecipitation (CoIP) was performed with the antibody specific for the HA peptide. Precipitated proteins were separated by SDS-PAGE. Immunoreactions were performed with the antibody specific for LHCA1 (22 kD), followed by incubation with the antibody directed towards LHCA2 (23 kD), LHCA3 (25 kD), LHCA4 (22 kD), and LHCB1 (20 kD) as control; (**C**) Confirmation of the interaction of WHY1 and LHCA1 by bimolecular fluorescence complementation assays (BiFC). The onion epidermal cells were transiently transformed with the full length WHY1 and the full length LHCA1. Constructs were fused to either c-myc-GFPn173 or HA-GFPc155 and vice versa. The WHY1 and LHCA1 fused to full length GFP and empty vector were used as controls. All constructs were under the control of the 35S promoter. Fluorescence images are shown on the upside and bright field images are shown on the underside, respectively. C1: WHY1-GFPn173 + LHCA1-GFPc155; C2: LHCA1-GFPn173 + WHY1-GFPc155; C3: WHY1-GFP; C4: LHCA1-GFP; C5: WHY1-GFPn173 + LHCA4-GFPc155; C1-2: scale bar 21 μm; C3-5: scale bar 100 μm.

In order to know whether pWHY1 affects the function and activity of photosystem I on protein level, we searched for the WHY1 interacting protein library [28]. Interestingly, LHCA1, which has a molecular weight of 22 kDa, was identified as one of the putative interaction partners (Figure 3A). LHCA1 is one of the four subunits of the peripheral light-harvesting complex (LHCI) in photosystem I (PSI) [29]. The interaction between LHCA1 and WHY1 was confirmed by fresh retransformation of yeast two hybrid (Figure 3A).

Next, we conducted a co-immunoprecipitation assay using the three-solubilized thylakoid membrane fractions (Figure S1) as inputs and the antibody against HA-tag as the binding partner. Immunoblotting using antibodies against LHCA1, LHCA2, LHCA3, LHCA4, and the photosystem II LHCB1, respectively, showed that these proteins were all present in the Fraction 3, whereas only LHCA1 was detectable in that of the co-IP samples, indicating a direct interaction between WHY1 and LHCA1. No interactions between WHY1 and the others LHCAs were discovered (Figure 3B). Here, the photosystem II protein LHCB1 was used as a separation control and it gave signals in both Fraction 2 and 1 in the input gel. A weak signal of LHCB1 in the lane of Fraction 3 of the same gel might hint a slight contamination of photosystem II. Similarly, the appearance of a weak signal of LHCA3 and an even-weaker signal of LHCA4 in the lane of Fraction 2 of the input gel probably arose from cross-contamination during sucrose gradient ultracentrifugation that were normally difficult to be avoided [26].

Further, a bimolecular fluorescence complementation (BiFC) assay was performed in onion epidermal cells to investigate the interaction between WHY1 and LHCA1 in vivo. WHY1 and LHCA1 were fused to YN vector pHA-GFPn173c or YC vector pcMYC-GFPc155c. As shown in Figure 3C, co-expression of WHY1-HA-GFPn173c and LHCA1-MYC-GFPc155c reconstituted a functional GFP in onion epidermal cells, and co-expression of WHY1-MYC-GFPc155c and LHCA1-HA-GFPn173c also reconstituted a functional GFP in onion epidermal cells. The co-expression of LHCA4 and WHY1 served as a negative control, which did not show the fluorescent signals (Figure 3C). The expression of LHCA4 and WHY1 in onion epidermal cells was detected by immunodetection (Figure S2). Fusions of WHY1 or LHCA1 to the full length GFP confirmed their plastid localization (Figure 3C). These results suggest that WHY1 interacts with LHCA1 both in vitro and in vivo. We here confirmed the interaction of WHY1 and LHCA1 is a part of the photosynthetic apparatus, implying that WHY1 might be involved in the photosynthetic apparatus complex formation on the protein level. However, native LHCA1 forms functional dimer with LHCA4 [30], which per se did not interact with WHY1 in these assays (Figure 3). Taken together, these results suggest that WHY1 interacts with LHCA1 in vitro and in vivo. Considering the roles of LHCA1 in photosynthesis and the similarity of photosynthesis parameters in *WHY1* mutants and *lhca1*, the interaction of WHY1 and LHCA1 may have a function in regulating photosynthetic activity.

### 2.4. WHY1 Affects the Expression of Plastid NDH Genes

**Figure 4:**
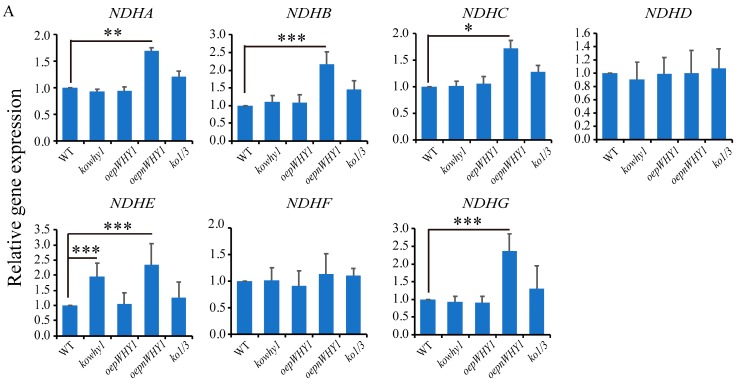

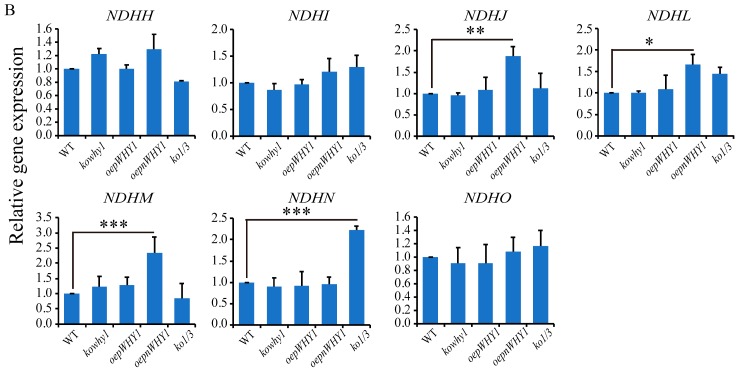
Expression of NDH-encoded genes in different *WHY1* mutants and wild-type plants. (**A**) Expression analysis of NDH membrane complex encoded genes in *WHY1* mutants and wild-type plants by qPCR; (**B**) Expression analysis of NDH subcomplex-A encoded genes in *WHY1* mutants and wild-type plants. The transcript level in each case was normalized to that of *GAPC2* as a reference gene, and the expression level of WT was set as 1. Three biological replications were used in the analysis. Asterisk indicate significant differences in gene expression compared to WT (Tukey–Kramer method, * *p* < 0.05, ** *p* < 0.01, *** *p* < 0.001).

The ETR and Y(I) was increased, but Y(II) was steady in *kowhy1* mutant, suggesting that the up-regulation of cyclic electron flow around PSI in *kowhy1* mutant led to the increase of PS I activity [24]. NDH is a large multi-protein complex containing more than 30 subunits which are encoded by both nucleus and organelle genomes. It has a key function in one of the CET (cyclic electron transport around photosystem I) pathways [31]. We then examined whether NDH subunits of membrane subcomplex and subcomplex A encoded by organelle genomes were affected by WHY1. qPCR analysis showed that the expression level of *NDHA*, *NDHB*, *NDHC*, *NDHE*, *NDHG*, and *NDHJ* were increased by more than 1.5-fold in the *oepnWHY1* mutant, while the transcripts of *NDHD*, *NDHF*, *NDHH*, and *NDHI* were almost invariable (Figure 4). The expression of these genes was steady in both *oepWHY1* and *ko1/3* mutants while the expression of *NDHE* was slightly increased in *kowhy1* mutant (Figure 4). These results raise the possibility that WHY1 may affect the activity or the formation of the NDH complex by regulating NDH gene expression.

### 2.5. NDH Activity and the Accumulation of NDH18 Were Changed in WHY1 Mutants

**Figure 5:**
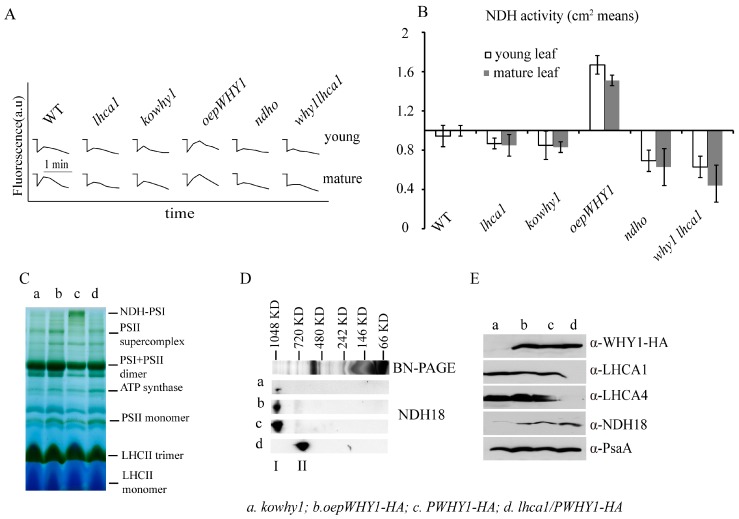
The stability of NDH-PSI complexes of thylakoid membranes and NDH activities in the different *WHY1* and *lhca1* mutants. (**A**) Determination of NDH activity by chlorophyll fluorescence analysis. Five-week-old plants including different *WHY1* and *lhca1* mutants, *why1lhca1* double mutant and wild-type (WT) were illuminated for 5 min with AL (200 μmol m^−2^ s^−1^). After illumination, the subsequent transient increase in chlorophyll fluorescence were recorded as an indicator of NDH activity (a.u.; arbitrary units). The fluorescence levels were standardized by the Fm level. Curves shows trace of chlorophyll fluorescence in mutants and WT plants. *ndho* line was used as positive control; (**B**) Quantification of NDH activity measured in (**A**). The transient increase in chlorophyll fluorescence in mutants was quantified by comparing the peak area with that of WT. The peak area of WT was set as 1. Data presented are the mean of ± SE (*n* = 5–6); (**C**) Blue Native PAGE of thylakoid membrane, Band I is the NDH-PSI supercomplex detected in *PWHY-HA* line; (**D**) 2D SDS-PAGE separation and immunodetection of NDH-PSI supercomplex containing NDH18 protein (I) and monomer NDH18 (II); (**E**) Immunodetection of thylakoid membrane, the antibody against to HA peptide, LHCA1, LHCA4, NDH18 (provided by Shikanai group, Japan) and PsaA in *kowhy1* (a), *oepWHY1-HA* (b), *PWHY1-HA* (c), and *PWHY1-HA* in *lhca1* background, *lhca1/PWHY1-HA* (d).

To further examine the effect of WHY1 on NDH activity, in vivo measurement of NDH activity was applied in *lhca1*, *kowhy1*, *lhca1why1*, *ndho*, and *oepWHY1* mutants and wild-type plants. *ndho* mutant lacked the peptides encoded by the nuclear gene *NDHO* (*At1g74880*) [32]. A post illumination rise in chlorophyll fluorescence of five-week-old rosette leaves was determined by using a PAM chlorophyll fluorometer. After illumination for 5 min with actinic light (ACT; 200 μmol m^−2^ s^−1^), transient increases in chlorophyll fluorescence in dark were recorded as an indicator of NDH activity (Figure 5A, see Section 4). As showed in Figure 5, absence of NDHO leads to a loss of NDH activity. Moreover, NDH activities were declined in *lhca1*, *kowhy1*, and *lhca1why1* mutants, but increased in *oepWHY1* line compared to wild-type (Figure 5A,B). These results suggest that WHY1 and LHCA1 can affect NDH activity.

We next examined the association of WHY1 with NDH-PSI super complex. The blue-native polyacrylamide gel electrophoresis (BN-PAGE) of thylakoid membrane protein from *PWHY1-HA* in *lhca1* background (*lhca1/PWHY1-HA*), *kowhy1*, *oepWHY1-HA*, and *PWHY1-HA* mutants were conducted using the NDH18 protein (a NAD(P)H dehydrogenase) as a representation to detect the NDH-PSI supercomplex [33,34]. Surprisingly, the PSI+LHCI complex protein was declined in *kowhy1* and *lhca1* mutants (Figure 5C) and was only detected in *PWHY1-HA* line. By 2D gel, the result further showed that NDH protein accumulation was decreased in *kowhy1* mutant and the monomeric NDH was increased in *lhca1* mutant, and LHCA4 protein was disappeared in the *lhca1* mutant (Figure 5D,E). This indicated that loss of WHY1 blocked NDH18 accumulation while loss of LHCA1 and LHCA4 enhanced NDH18 and monomeric NDH accumulation. It might suggest that both WHY1 and LHCA1 affect the composition of NDH-PSI super complex and the super complex structure, but WHY1 or LHCA1 does not affect the same subunit of NDH-PSI.

### 2.6. WHIRLY Proteins Alter the Photosynthetic Performances after High Light Treatment

**Figure 6:**
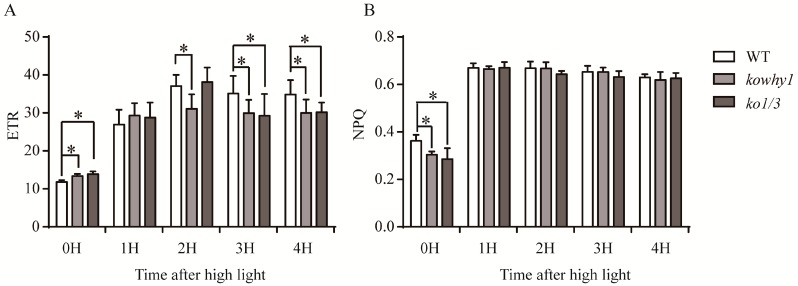
Measurement of photosynthetic parameters in *WHY* mutants and wild-type plants after high light treatment. (**A**) Electron transport rate (ETR) in *WHY* mutants and wild-type plants under high light; (**B**) Non-photochemical quenching (NPQ) in *WHY* mutants and wild-type plants under high light. Means ± SE of at least 10 independent measurements are shown. Asterisk indicate significant differences compared to WT (* *p* < 0.05, Student’s *t* test).

Cyclic electron flow around photosystem I (PSI) seems to prevent photoinhibition of photosystem I [35]. As reported in previous studies, WHY1 was involved in the alteration of ETR as well as gene expression, activity and formation of the NDH complex which both are related to cyclic electron transport. To further investigate the effects of WHY1 in photoinhibition, *kowhy1*, *ko1/3*, and wild-type plants grown for four weeks under normal light condition were transferred for 1, 2, 3, and 4 h to high light conditions (800 μmol m^−2^ s^−1^). Image-PAM was used to determine the chlorophyll fluorescence parameters. Under normal light conditions (80 μmol m^−2^ s^−1^), photochemical quenching (qP), quantum efficiency of PSII (ΦPSII) and maximum efficiency of PSII (Fv/Fm) were similar in wild-type plants and the mutants (*kowhy1* and *ko1/3*) (Figure S3). The non-photochemical quenching (qN and NPQ) were lower in *kowhy1* and *ko1/3* mutants compared to wild-type while the electron transport rate (ETR) was higher in these mutants (Figure 6 and Figure S3). Hence, the photosynthetic parameters were similar in both young and senescent leaves of *kowhy1* mutant (Figure 1 and Figure 6).

Under high light conditions, qP and ΦPSII were similarly decreased in all lines while NPQ and qN were increased. Therefore, the linear electron transport of photosynthesis was inhibited and the more excessive excitation energy was produced in all plants under high light conditions [36]. qP and ΦPSII were similar in *kowhy1*, *ko1/3*, and wild-type plants. Interestingly, NPQ and qN were also similar in *kowhy1* and *ko1/3* mutants compared to wild-type after high light treatment, while they were lower in *kowhy1* and *ko1/3* mutants compared to wild-type under normal light conditions. However, the ETR in *kowhy1* and *ko1/3* plants were lower than wild-type after 2 or 3 h high light treatments respectively (Figure 6), implying that electron transport of photosynthesis, especially the cyclic electron transport of Photosystem I, was inhibited in *WHY* mutants under high light conditions. Therefore, WHY1 may be involved in light adaption by regulating the electron transportation of photosynthesis.

### 2.7. WHIRLY Proteins Affect the Expression of Photosynthesis Related Genes under High Light Condition

**Figure 7:**
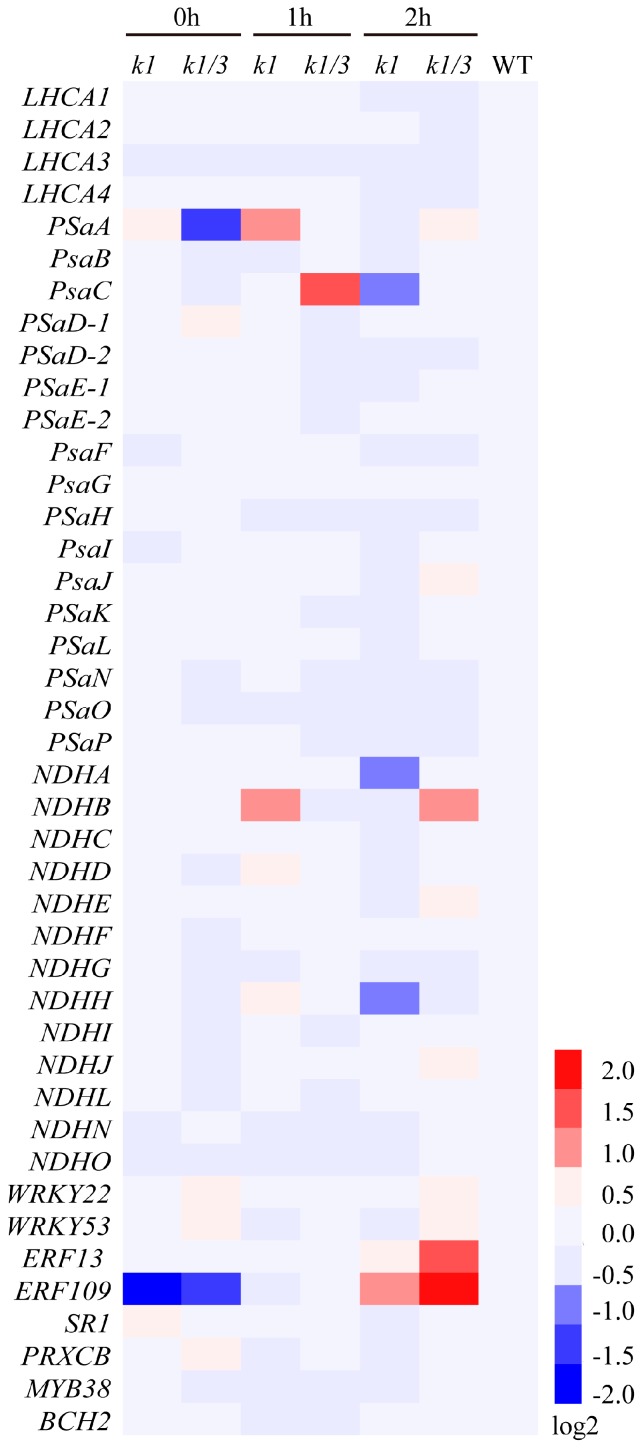
Heat map of gene expression in *kowhy1* and *ko1/3* mutants after high light treatment. Gene expression in *kowhy1*, *ko1/3* mutants after high light treatment was analyzed by qPCR. The transcript level in each case was normalized to that of *GAPC2* as a reference gene, and the expression level of WT was set as 1. Three biological replications were used to analyze. Heat map were drawn by Heml software used log2 of expression.

To further confirm the role of WHY1 in electron transport after high light treatment, we examined the expression of numerous genes encoding LHCI complex, PSI core complex, and NDH complex by qPCR. Under normal light condition, there were little differences in the expression levels of PSI-related genes between *kowhy1* and wild-type, while *PsaA* was down-regulated in *ko1/3* mutant (Figure 7). However, after high light treatment, the expression level of *PsaA* was higher in *kowhy1* than in wild-type, while they were similar in *ko1/3* and wild-type plants. Transcription level of *PsaC* was increased only in *ko1/3* line, which may imply a relationship in function between WHY1 and WHY3. PsaA and PsaB can form hydrophobic interaction site of the PSI core for efficient docking of the electron transfer donors [37]. *NDHB*, which has been reported to have a relation with CET in cyanobacterium [38], was up-regulated in *kowhy1* and *ko1/3* mutants (Figure 7). The rates of PSI cyclic electron transport in *ndhb* mutant of *Cyanobacterium Synechocystis* were decreased after dark treatment but increased after exposure to high light, compared to the wild-type [38]. *WRKY22* and *BCH2* (*BETA CAROTENOID HYDROXYLASE 2*), which were repressed and induced by high light, were used as the negative and positive control, respectively [39,40]. The expression levels of *WRKY22* and *BCH2* in *kowhy1* and *ko1/3* mutants were similar to wild-type under high light.

Further, mutation of *WHY1* led to the increased expression level of *ERF109* and *ERF13* compared to wild-type plants in response to high light treatment (Figure 7). ERF13 is a CE1 (coupling element 1) binding protein and confers ABA hypersensitivity in Arabidopsis [41]. ERF109 (RRTF) was induced by singlet oxygen and increased in response to JA [42,43]. ERF109 enhances the defense responses to high light (HL) stress in young leaves, while it induces senescence and chlorosis in older leaves [44]. Moreover, the expression of Calcineurin B-Like-Interacting Protein Kinase14 (CIPK14, also named SR1) and WHY1 targeted genes included *PRXCB* and *MYB38* were also detected [21,28]. The transcription level of *SR1* that interacts with and phosphorylates WHY1 to increase its accumulation in the nucleus was similar in *kowhy1*, *ko1/3*, and wild-type plants under high light conditions. Previous studies showed PRXCB is a peroxidase regulated by light [45] and MBY38 functions as a positive element in blue light signaling [46]. Their expression level was steady in *WHY* mutants and wild-type plants under high light conditions. Collectively, these findings suggest that WHY1 may affect PSI-NDH complex formation and is involved in the plant hormone response pathways during light adaptation.

## 3. Discussion

In chloroplasts, WHY1 were found to associate with nucleoids [14,15,47], but only a minor part of WHY1 colocalizes with cp nucleoids that was showed by high-resolution imaging [48]. The elusive and complex localization of WHIRLY may represent its multifunctionality. WHY1 is located at the boundary between thylakoids and nucleoids, where are attractive to a protein that may link the operation of the photosynthetic electron transport chain to gene expression [8]. In addition, *HvWHY1* RNAi mutants have delayed senescence phenotype under high light irradiation while that was not observed under normal light and dark. Therefore, WHY1 was proposed to closely associate with the components of thylakoid electron chain and is related to photosynthesis and the perception of light intensity [8]. However, the relationship of WHY1 and photosynthesis as well as light is not yet clear. The results of this study showed photosynthetic performance was affected in *WHY1* knock out mutant under different light conditions. Furthermore, WHY1 were found to interact with LHCA1 in chloroplasts and affect the expression of photosystem I-LHCI and NDH encoding genes, which were consistent with the alternation of NDH activity and structure in *kowhy1* mutant.

In this study, higher Y(I) in *kowhy1* mutant demonstrated an elevated redox rate of P700 caused by the lack of WHY1 at an early stage of leaf senescence (Figure 1). The lower NPQ and higher ETR implied that a proton gradient across the thylakoid membrane formed was affected in the *kowhy1* mutant [36]. However, the similar photochemical quantum yield of photosystem II Y(II) in *WHY1* mutants and wild-type plants showed linear electron transport of photosynthesis, which is related to photosystem II activity may not be affected by WHY1 [49]. The lower NPQ in *kowhy1* plants may be a mechanism to up-regulate photosynthetic electron transport [50]. Under high light conditions, in accordance with barley, the ETR in *Arabidopsis kowhy1* and *ko1/3* mutants was lower than in wild-type (Figure 6), while the NPQ were similar in *kowhy1*, *ko1/3* and wild-type plants. It is possible that self-protective machinery of *kowhy1* and *ko1/3* mutants, by dissipating the excessive excitation energy harmlessly as heat, is also operating under short-time high radiation instead of lowering NPQ to rescue the ETR inhibited in *kowhy1* and *ko1/3*. Therefore, WHY1 in chloroplasts may have a positive role in photosynthetic electron transport.

Mutants analysis of photosystem I subunits showed that deficiencies in photosynthesis promote the induction of leaf senescence [3]. Interestingly, overexpression of WHY1 in chloroplasts accelerated leaf senescence (Lin et al., 2017, unpublished data). Unexpectedly, overexpressing *plastid-localized WHY1* (*pWHY1*) does not alter the expression level of genes encoding the PSI core complex and PSI external antenna, and that of plastid genes encoding the NDH complex. However, WHY1 is able to physically interact with LHCA1 (Figure 2), which belongs to the light harvesting complex of photosystem I [30]. It is just consistent with the phenomena that the WHIRLY protein was not detected in all cp nucleoids and punctate immunofluorescence signals were also observed in extra-nucleoid (stroma) regions in the zygote of *C. reinhardtii*, *K. flaccidum*, and *M. Polymorpha* plants [48]. LHCA1 was located closely to PsaG of the core complex and proposed serving as an anchor for the binding of the whole LHCI to one side of the PSI core complex [29,30]. Specific location of each LHCA protein within the PSI-LHCI super complex is important to excitation energy trapping [51]. A previous study showed that an *Arabidopsis thaliana* Phloem Intercalated with Xylem-like 1 (AtPXL1) protein interacts with and phosphorylates LHCA1 in the regulation of signal transduction pathways under temperature fluctuations [52]. Thereby, we hypothesize that the interaction of WHY1 and LHCA1 may have effects on the structure and function of the photosynthetic apparatus and is involved in the signal transduction between chloroplasts and nucleus during leaf development and responds to various stresses. Alternatively, WHY1 in chloroplast may have a protective effect on photosystem I that was similar to the function of PsaG, which changes the conformation of PSI and thereby allows higher electron transfer under high light or other photoinhibitory conditions [53].

In addition, overexpression of WHY1 in both nucleus and plastid improved the transcription level of a number of genes encoding PSI core subunits which are essential to the formation of super complexes of PSI [27,37] (Figure 1B) and multiple NDH genes encoded by the plastid genome (Figure 4). However, the expression of those genes was not affected by overexpression of *pWHY1* in plastids. Hence, WHY1 might not regulate the NDH-PSI super complex genes directly in transcriptional level, but have a role in the formation of NDH-PSI super complex. The formation of the NDH-PSI super complex is necessary for the stability of NDH, especially under high-light conditions [34]. NDH complexes contain homologs of the subunits of mitochondria complex I and eubacterial NADH dehydrogenase and are also equipped with different “catalytic” domains, suggesting that they probably utilize different substrates as electron donors [34]. Loss of WHY1 and LHCA1 leads to the decline of NDH activity (Figure 5A) and inhibit NDH-PSI supercomplex accumulation (Figure 5D,E). Therefore, WHY1 protein located in chloroplasts and interaction with LHCA1 may facilitate the stability of the NDH-PSI supercomplex. Moreover, WHY1 protein may act as a coordinator between chloroplasts and nucleus and affect the NDH-dependent cyclic electron transport around photosystem I in a manner depending on hormone signal response since the expression of several hormone-related genes was varied in *WHY1* mutated plants (Figure 7) [17].

## 4. Materials and Methods

### 4.1. Plant Materials and Growth Conditions

All plants used in this study were *Arabidopsis thaliana* (L.) Heynold ecotype Columbia 0 background. The *WHY1* knock-out line (*kowhy1*) that has a T-DNA insertion in exon1 (*SALK_023713*) and the *LHCA1* knock-out line that T-DNA inserted in the first exon (*lhca1*, *SAIL_870_E09*) were received from NASC (Nottingham, UK). The homozygous line was selected by PCR using the primers provided by the Salk Institute (http://signal.salk.edu/) [54]. *WHY1* lines including *oepnWHY1*, *oepWHY1*, *Pwhy1* were prepared from the former work [21]. The WT*/why1* line was the *kowhy1* line crossing with wild-type plants and the double *why1lhca1* mutant was the *kowhy1* line crossing with the *lhca1* line, and they were identified by qRT-PCR. Seeds of *WHY1* and *WHY3* double mutant (*ko1/3*) were kindly provided by Prof. Normand Brisson, Department of Biochemistry, Montreal University, Montreal, Canada. Seeds of these mutants and wild-type were grown in a growth chamber at 23 °C with 13 h light condition (100 μmol m^−2^ s^−1^). For high light treatment, seeds of *kowhy1*, *ko1/3* and wild-type were grown for 4 weeks at 23 °C with 13 h light condition (80 μmol m^−2^ s^−1^) and then transferred to high light conditions (800 μmol m^−2^ s^−1^) for 1, 2, 3, and 4 h.

### 4.2. Analysis of Photosynthetic Parameters

Seven-week-old *Arabidopsis* wild-type plants and the T-DNA insertion mutant of *At-WHY1* were grown in a controlled growth chamber at a photon irradiance of 100 μmol m^−2^ s^−1^. A Dual PAM 100 measuring system (Walz, Effeltrich, Germany) with a Dual-DR and a Dual-E measuring head was used for simultaneous measurement of chlorophyll fluorescence and P700 oxidation. Two leaves of each plant and five plants of each line were used for the measurements. According to the method described in http://www.walz.com/products/chl_p700/dual-pam-100/applications.html, experiments were carried out by using the automated Induction and Recovery Curve routine provided by the Dual-PAM software, with repetitive application of saturation pulses (SP) for assessment of fluorescence and P700 parameters, from which the quantum yields of PS I and PS II were derived by the software. The measuring mode was set up for P700 and fluorescence analyses mode is SP analysis, induction curve was recorded, light intensity 125 μmol m^−2^ s^−1^, total collection time 6 min, SP duration time is 5 min. The max data of Y(I), Y(II), and Y(NPQ) were used deduced form the data collected. Initial parameters of Photosystem II fluorescence including dark fluorescence yield (F_0_) and maximal fluorescence yield (F_m_) in plants were measured after dark adaptation for 15 min The calculation of quantum yields of Photosystem I Y(I), Photosystem II Y(II) and non-photochemical quenching of photosystem II fluorescence (NPQ) were performed with equations [55,56]. Electron transport rates (ETR) were calculated and recorded by the Dual-PAM software [57,58].

For high light treatment, plants were transferred from normal light condition (80 μmol m^−2^ s^−1^) to high light conditions (800 μmol m^−2^ s^−1^) for 1, 2, 3, and 4 h. The chlorophyll fluorescence parameters in high light treatment were measured using IMAGING-PAM MAXI (Heinz Walz GmbH, Effeltrich, Germany) after dark-adaptation for 15 min as described by Shao [59]. Fully expanded leaves were selected for measurement and were placed onto a block with wet blotting paper. An area of interest with a diameter of 1 cm was selected in the middle of the whole leaf to record chlorophyll fluorescence parameters. Data of the maximum quantum yield of PSII (Fv/Fm) that represents the maximum quantum yield of PSII, the quantum yield of PSII (ΦPSII), photochemical fluorescence quenching coefficients in PSII (qP) that is a measure of the overall openness of the reaction center, non-photochemical quenching (NPQ and qN), and ETR were exported from IMAGING-PAM software. Photochemical (qP) and no-photochemical quenching (NPQ, qN) are protective mechanisms to photosynthetic machinery via balancing light utilization [24].

### 4.3. Quantitative Real-Time PCR

Total RNA was isolated according to the manufacturer’s protocol of TransZol UP (TransGen Biotech, Beijing, China). First-strand cDNA was generated from 1μg of total RNA using TranScript one-step gDNA removal and cDNA synthesis supermix kit (AT311, TRANSGEN), following the instruction. qPCR was performed to analyze the expression of genes in the CFX96 machine (Bio-Rad Company, Hercules, CA, USA) in a whole volume of 15 μL, including 2 μL of 2× UltraSYBR Mixture (cw0956c, CoWin Biosciences, Beijing, China), 0.5 μL of each gene-specific primer (10 μm). To determine the relative expression rate, data were normalized to the expression level of wild-type or in five-week-old plants (which were set to 1) after normalized to the internal *GAPC2* gene control. Three technical replicates of three biological replicates and the determination of a melting curve of the amplified PCR products were carried out. Primers used for qPCR were listed in Table S1 (In Supplementary).

### 4.4. Yeast Two-Hybrid System Screen and Confirmation

The activation domain of the full length WHY1 cDNA was deleted, and the truncated cDNA was cloned into the bait pGBKT7 vector, and screened the cDNA expression library that was prepared from five-week-old rosette leaves of Arabidopsis according to the method described by Miao and Zentgraf [60]. The two-hybrid assays were performed as described in Clontech’s Matchmaker GAL4 Two-Hybrid System 3 manual. *WHY1* and *LHCA1* with full-length cDNA sequences were cloned into prey vectors. The truncated version of *WHY1* lacking the activated domain and full-length cDNA sequences encoding LHCA1 were inserted in the bait construct, respectively. These recombinant plasmids were introduced into yeast strain Y187 containing *LacZ* gene. X-gal agarose overlay assay were performed according to the protocol (http://biochemistry.ucsf.edu/~herskowitz/protocols.html) using *o*-nitrophenyl β-galactopyranoside as substrate.

### 4.5. Immunological Analyses

Proteins were extracted from rosette leaves according to Miao et al. [61]. Equal amounts of proteins (8–25 μg) determined according to Bradford method [62] were subjected to SDS-PAGE on 14% (*w*/*v*) polyacrylamide gels [63], and separated proteins were transferred to nitrocellulose by semi-dry electroblotting [61]. After incubation with the primary antibody and secondary antiserum in turn, immunoreactions were detected with a chemiluminescent substrate (GE Healthcare, Chalfont St. Giles, Buckinghamshire, UK). Primary antibodies directed toward the HA-tag oligopeptides (Sigma, Munich, Germany) and the 14kD histone H2B (Cell Signaling, Munich, Germany), all other antibodies were purchased from Agrisera, Sweden. GFP specific antibody was purchased from Roche Diagnostics GmbH Germany (Mannheim, Germany).

### 4.6. Coimmunoprecipitation of Photosystem I Complex Proteins

A crude fraction of chloroplasts was prepared from the seventh leaves of 5-week-old Arabidopsis plants referred to the method of Poulsen [64]. Plastids were osmotically lysed and fractions were obtained after centrifugation at 20,000× *g* for 10 min. Thylakoids employed for isolation of super molecular complexes were prepared and stored at −80 °C in buffer with 50% (*v*/*v*) glycerol [65,66]. After solubilization of thylakoids by 0.02% or 0.5% (*w*/*v*) dodecyl-α-d-maltoside [67,68], complexes were separated on continuous 0.2–1.0 M sucrose density gradients containing 0.02% or 0.5% dodecyl-α-d-maltoside and 10 mM Hepes by centrifugation in a Beckman (Indianapolis, IN, USA) Ti 41.14 rotor for 24 h at 200,000× *g* at 4 °C.

The photosystem I containing thylakoid membrane fractions were incubated with the HA-tag peptide antibody at 4 °C for one hour on a rotating wheel. After the addition of protein A beads, the mixture was incubated likewise for one hour and then centrifuged at 7000 rpm for 20 s. Protein A beads were washed three times with buffer (50 mM Tris-HCl including 0.1% nonidet P-40, pH 8.0) and then resuspended in SDS-PAGE loading buffer.

### 4.7. Bimolecular Fluorescence Complementation Assay (BiFC)

The plasmid of the full length AtWHY1 sequence fused to GFP was available [69]. The full-length AtLHCA1 coding sequence was amplified by PCR from NASC cDNA clone U12182 and was cloned in frame in front of the GFP coding sequence using the binary gateway vector pBatTL-B.GFP2 containing a double 35S promoter. The full length AtWHY1 and AtLHCA1 cDNA were cloned to pHA-GFPn173c vector and pcMYC-GFPc155c vector via *XbaI*/*KpnI*, respectively [60]. Both appropriate plasmids were introduced to onion epidermal cells with the biolistic bombardment method [69]. GFP-dependent fluorescence was observed using an epifluorescence microscope (Axiophot, Zeiss, Jena, Germany) and a Leica SP8 confocal laser scanning microscope (Leica, Heidelberg, Germany). For immunological analysis of proteins, 200 mg of onion epidermal tissue were extracted 24–48 h after transformation.

### 4.8. In Vivo Measurements of NDH Activity

NDH activity of five-week-old rosette leaves from different mutants and WT were measured using a PAM chlorophyll fluorometer according to Peng (2009) [33]. The plants were illuminated for 5 min with actinic light (ACT; 200 μmol m^−2^ s^−1^). After illumination, the fluorescence rise of the F_0_ level due to electron donation to PQ via NDH in dark after switching off the actinic light is taken as a measure of the NDH activity, which was monitored as the subsequent transient increase in chlorophyll fluorescence [33,70,71,72]. The transient increase in chlorophyll fluorescence in mutants was quantified by comparing the peak area with that of WT during 1 min. The peak area of WT was set as 1.

### 4.9. Separation of Photosynthetic Complexes by Blue-Native Polyacrylamide Gel Electrophoresis (BN-PAGE)

Leaf samples were immediately frozen by liquid nitrogen after harvest. Thylakoid membranes were isolated at 4 °C according to Li et al. (2010), and BN-PAGE and 2D SDS-PAGE were performed as previously described [73]. An equal amount of supernatant equivalent to 10 μg of Chls was subjected to BN-PAGE with a 4–20% gradient gel. Electrophoresis was carried out for 3 h at 30 mA at 4 °C.

## Supplementary Materials

Supplementary materials can be found at www.mdpi.com/1422-0067/18/11/2352/s1.
