# Supplementary material for: Dual-Located WHIRLY1 Interacting with LHCA1 Alters Photochemical Activities of Photosystem I and Is Involved in Light Adaptation in Arabidopsis
Source: Int J Mol Sci. 2017 Nov 7;18(11):2352. doi: 10.3390/ijms18112352 (PMC5713321; doi:10.3390/ijms18112352)
Supplement: Supplementary file 1 [file ijms-18-02352-s001.pdf]

Supplementary Materials: Dual-Located WHIRLY1 Interacting with LHCA1 Alters Photochemical Activities of Photosystem I and is Involved in Light Adaptation in Arabidopsis

Dongmei Huang, Wenfang Lin, Ban Deng, Yujun Ren, and Ying Miao

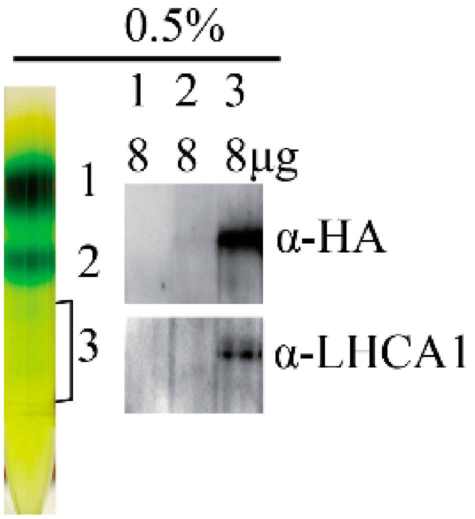

Figure S1. Sucrose density gradient ultracentrifugation of pigment protein complexes prepared from thylakoids solublized by 0.5% dodecyl-α- D-maltoside. Band 1 consists of free pigments. Band 2 contains LHCII and photosystem II complexes. Band 3 contains photosystem I complexes. Proteins from fractions 1–3 were used for immunological analysis. The blot was immunodecorated with the HA-tag antibody, followed by immunodecoration with an antibody specific for the LHCA1 (22 kD).

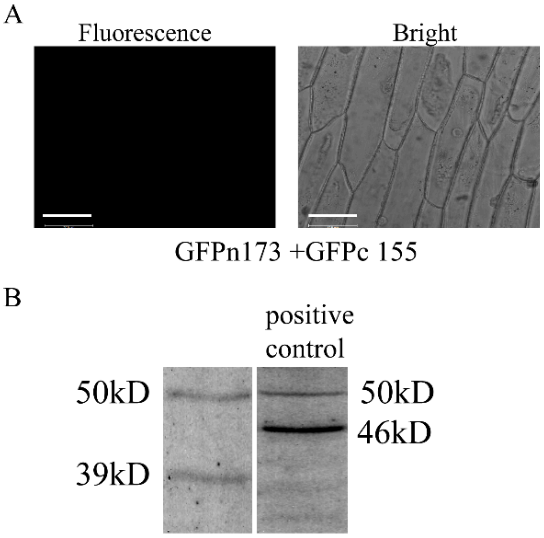

Figure S2. Negative controls for interaction of WHY1 and LHCA1. (**A**) Co-expression GFPn173 and GFPc155 without any fusions in onion epidermal cells. Fluorescence images are shown on the left and bright field images are shown on the right side, respectively. Scale bar 77.6 μm. (**B**) Immunodetection of the constructs (WHY1-GFPn173+LHCA4-GFPc155) or positive control (WHY1-GFPn173+WHY3-GFPc155) expression in the cells with the antibody against the full length GFP.

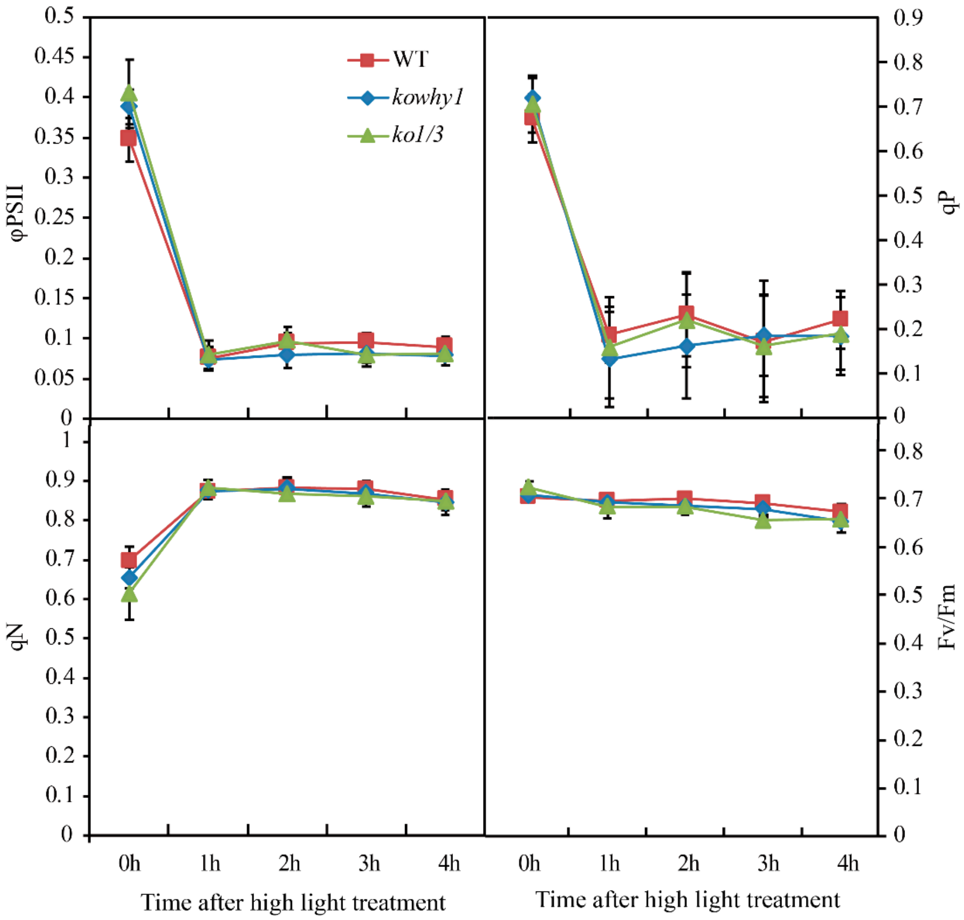

Figure S3. Photosynthetic parameters of *WHY1* mutants after high light treatment. Quantum efficiency of PSII(ΦPSII); photochemical quenching (qP); non-photochemical quenching(qN); maximum efficiency of PSII (Fv/Fm) in *WHY1* mutants under high light for 1, 2, 3, 4 hours. Means and SD of at least 10 independent measurements are shown.

Table S1. Primers for quantitative PCR.

TAIR number	Gene Name	Primer sequence (5'-3')
AT3G54890	*LHCA1*	FP:GCCGGGAATGTTGGTCGTATCAG<br>RP:CAAACCCAAAGTCACCAGGAGCAG
AT3G61470	*LHCA2*	FP:GTCTTGAAGGTCAACAACGTCGAG<br>RP:ACCACCCAAGAAACTTGGAGAAG
AT1G61520	*LHCA3*	FP:AAACCAGTTGCGTCTGCCTCAC<br>RP:ATGGTCTGTTGGCTCCTTGCTTG
AT3G47470	*LHCA4*	FP:CCGCTTAACTTTGCTCCTACGC<br>RP:AACATCGCCAACCTCCCGTTTG
AT1G45474	*LHCA5*	FP:AGAGCTTGTTCACTCTCGTTTCGC<br>RP:TGTGGTCCGAAGGAGATCAGTG
AT1G19150	*LHCA6*	FP:GGTTTCGATCCTCTCGGTTTAGGG<br>RP:ACCTAACCGCTCGAGACATTCTG
ATCG00350	*PsaA*	FP:AGCGAGCACCAGTTTGACTTGG<br>RP:AAGCTACTTTGCCACCCACTGC

ATCG00340	*PsaB*	FP:AATTTCCTGGCGTGGTTATTGGC<br>RP:CGTTCATGAGCCCACGCTAAAG
ATCG01060	*PSAC*	FP:TTCTGCTCCAAGAACCGAGGAC<br>RP:GTTGGACAGGCGGATTCACATC
AT4G02770	*PSAD-1*	FP:AACACACCGTCTCCGATCTTCG<br>RP:ACTCTTCCACTTGCGCTTTACG
AT1G03130	*PSaD-2*	FP:AGCCGTGAAAGAAGCTCCAGTTG<br>RP:CTTCCGGCGAAGATTGGTGATG
AT4G28750	*PSaE-1*	FP:AGTTGTTGCCGTTGATCAGGAC<br>RP:CCCATTTAAGCTGCAACTTCTTCG
AT2G20260	*PSaE-2*	FP:AGTTGTGGCCGTTGATCAGGAC<br>RP:TTCTTCCACCTCGTCCAATGCG
AT1G31330	*PsaF*	FP:CTCTTGCTCTCAATGCTCAGATCG<br>RP:ACTTCCCGTAGTTGTCGAACCTG
AT1G55670	*PsaG*	FP:CCATCTCATTCCATGGCCTTCGTC<br>RP:TCCCGGTGGTTGATAGTTTGGG
AT3G16140	*PsaH*	FP:ACCCAAATCCATCCGGGCTAATG<br>RP:AGAGCCGTATACGTCCCATTGAC
ATCG00510	*PsaI*	FP:TGACAACTTTCAATAACTTACCCTC<br>RP:CCATTGCAATTGCCGGAAAGA
ATCG00630	*PSAJ*	FP:ATGGTTCGGTTCGTTAGCAG<br>RP:GGGAAATGTTAATGCATCTGGAA
AT1G30380	*PsaK*	FP:GGAGCTTTGGGTGCAAAGTGTG<br>RP:GGTCGTCGACGTTACCATTATCAG
AT4G12800	*PsaL*	FP:CCGAGCTGTTAAATCCGACAAGAC<br>RP:AAGGTACCACGCGATCAATGGG
AT5G64040	*PsaN*	FP:GCCTGTGATCAAAGCTCAACGC<br>RP:AAACCATGGCTGATCGTCTTCC
AT1G08380	*PsaO*	FP:CCGCTAAAGCCACTGCTTACTGTC<br>RP:TAGTAGCTCTCGTCACAACGTTCC
AT2G46820	*PsaP*	FP:CCAACATTAAGCGCCAAGAACCC<br>RP:TCAACCAGTTCCTCTCAAAGCAAG
ATCG01100	*NDHA*	FP:GGATGGAATTTGTGGCGTCAACC<br>RP:ACGGTAACCTCTCGCATTCTGC
ATCG00890	*NDHB.1*	FP:CGGGGGAGAGATTGAGCTTC<br>RP:TGGAGTGGGAGATCCTTCGT
ATCG00440	*NDHC*	FP:CAATTAGGAAGGGGCCGGAG<br>RP:TTGTAACCAAGCATCCCCGA
ATCG01050	*NDHD*	FP:TTTCTCCTTGGTTGTTGGTAGTCG<br>RP:TGACCGGGAGATGTTGAAGCTG
ATCG01070	*NDHE*	FP:TGGTTAGGGCTCTTATGTGTCT<br>RP:CAGCGGCTGCAATTGCTATAA

ATCG01010	*NDHF*	FP:CCCTTTCATTCCACTTCCAGTACC<br>RP:AAAGTCCACATGCGGCGAAG
ATCG01080	*NDHG*	FP:CGATACGTCATGGTACGGGG<br>RP:CGAGCCACAGAAATTGCACC
ATCG01110	*NDHH*	FP:ATTCGAAGCTGCCACCGGTATG<br>RP:TCAGCCGCTATTCCTCCAATACG
ATCG01090	*NDHI*	FP:TGGTCAACAAACCCTACGAGCTG<br>RP:AATGGATTCGACCGCGGAAACG
ATCG00420	*NDHJ*	FP:TAGCCATCCACGACTGAAACGG<br>RP:ACGTAAAGGCCACCCTATCCAAC
AT1G70760	*NDHL*	FP:ACAATCTTGGCAGCTCAACTCG<br>RP:TGCTAAGGCTGGATGGTCAATCG
AT4G37925	*NDHM*	FP: AGCTGAACGTGAAGAGCAAGGG<br>RP:TGACGTGTGACACTGCTTAGCC
AT5G58260	*NDHN*	FP:TATGGGTCCTCGAAGCAAAGGTC<br>RP:GGACGAAGAGAGGGAAGTAAAGCG
AT1G74880	*NDHO*	FP:GACAGGAGAGTATGCACTTGTTGG<br>RP:AGCATATCTGTTGGGAGCCAAGC
AT3G27690	*LHCB2*	FP:AGCAATGGCCACTTCAGCAA<br>RP:CGGAGGAGATCGTTGGATGG
AT4G28660	*PSB28*	FP:TTCAGTCGACGGATGTGAACGC<br>RP:TGTGCTTCGCCACAATCCCTTC
AT3G01440	*PNSL3*	FP:AAGCTTGAAGATGCAGCTAAGACG<br>RP:ACCGAACCAGGATCATGCCATTC
AT1G70580	*AOAT2*	FP:AACAAGCCGGAAAAGTCCCT<br>RP:CTGCTGGCAGAATTGTTGTCC
AT4G26530	*ATFBA5*	FP:AGGCACTCTGCTTAAACCGAAC<br>RP:TATCACTTCCGGTGCAACCTTTG
AT2G05100	*LHCB2.1*	FP:CAACGCCTGGTCTTACGCTA<br>RP:AACAGGGTGGTGTGGTTCA
AT5G52570	*BCH II*	FP:CCTGGTCTCTGCTTCGGTG<br>RP:TGTGTGGTGTAGCTGGTGAG
AT2G36890	*MYB38*	FP:CCGTATAACCCATCAACAAACCA<br>RP:GTTGTTGGCCCATCATTGTCA
AT3G49120	*PRXCB peroxidase*	FP:ACAATCTGTCACTTTGGCAGGAG<br>RP:TGCATTAGCGAGTTCCAGGAATG
AT5G01820	*SR1*	FP:TAGACACGAATCCGCAGACGAG<br>RP:TCGTCGTAGCCCTGTTTGAACC
AT 2G44840	*ERF13*	FP: AGCGACTTGCCGTTAAGTGTCG<br>RP: AACGGCATCACGGAGAGTGTTG
AT4G34410	*ERF109*	FP: TGGGCCAAGGGCTAAACTCAAC<br>RP: CAGCAGCAACAGGAGATGAAACTG

AT4G01250	*WRKY22*	FP: ACGGCGGAGCATAATCATCCAG<br>RP: TGGTTTCTGACGTGTGCTTCCG
